# Novel regulatory T cells controlling antibody production and systemic autoimmunity

**DOI:** 10.1186/ar3586

**Published:** 2012-02-09

**Authors:** Kazuhiko Yamamoto, Tomohisa Okamura, Keishi Fujio

**Affiliations:** 1 Department of Allergy and Rheumatology, The University of Tokyo Graduate School of Medicine, Tokyo, Japan

## 

Regulatory T cells (Tregs) are engaged in the maintenance of immunological self-tolerance and immune homeostasis. IL-10 has an important role in maintaining the normal immune state. We showed that IL-10-secreting Tregs can be delineated in normal mice as CD4^+^CD25^-^Foxp3^- ^T cells that express lymphocyte activation gene-3 (LAG-3), an MHC class II-binding CD4 homolog. CD4^+^CD25^-^LAG3^+ ^Tregs characteristically express early growth response gene-2 (Egr-2), a key molecule for anergy induction. Retroviral gene transfer of Egr-2 converts naïve CD4^+ ^T cells into IL-10-secreting and LAG-3-expressing Tregs. Moreover, CD4^+^CD25^-^LAG3^+ ^Tregs show B cell-dependent development. CD4^+^CD25^-^LAG3^+ ^Tregs, but not CD4^+^CD25^+ ^Tregs, strongly suppressed the antibody production in B cells co-cultured with helper T cells. Thus, IL-10-secreting Egr-2^+^LAG3^+^CD4^+ ^Tregs are closely related to B cells and can be exploited for the treat ment of autoimmune diseases.

Systemic lupus erythematosus (SLE) is a multisystem chronic inflammatory disease that affects many organs, and the immunological disorders are accompanied by autoantibody production. Recent case-control association study revealed that polymorphisms in the Egr-2 influence SLE susceptibility in humans. Interestingly, adoptive transfer of CD4^+^CD25^-^LAG3^+ ^Tregs from MRL/+ mice suppressed autoantibody production and the progression of nephritis in MRL/*lpr *lupus prone mice. In contrast, CD4^+^CD25^+ ^Tregs from MRL/+ mice exhibited no significant therapeutic effect upon transfer to MRL/*lpr *mice. These results indicate that CD4^+^CD25^-^LAG3^+ ^Tregs play key roles in the regulation of humoral immunity by the strong suppressive activity for B cell antibody production.

